# Tunable emission from green to red in the GdSr_2_AlO_5_:Tb^3+^,Eu^3+^ phosphor *via* efficient energy transfer

**DOI:** 10.1039/c7ra12260h

**Published:** 2018-01-17

**Authors:** Yu Zhang, Xuejie Zhang, Haoran Zhang, Lingling Zheng, Yuan Zeng, Yu Lin, Yingliang Liu, Bingfu Lei

**Affiliations:** Guangdong Provincial Engineering Technology Research Center for Optical Agricultural, College of Materials and Energy, South China Agriculture University Guangzhou 510642 P. R. China tleibf@scau.edu.cn

## Abstract

Herein, a series of GdSr_2_AlO_5_:Tb^3+^,Eu^3+^ phosphors were successfully synthesized through a high temperature solid-state reaction, and their crystal structures as well as photoluminescence properties were investigated in detail. Compared to the intense emission of ^5^D_0_ → ^7^F_1_ or ^5^D_0_ → ^7^F_2_ transition of Eu^3+^, another strong emission corresponding to ^5^D_0_ → ^7^F_4_ was observed. Concentration quenching is not obvious in Tb^3+^ or Eu^3+^-doped GdSr_2_AlO_5_ because structure isolation and energy transfer (ET) of Gd^3+^ → Eu^3+^ and Gd^3+^ → Tb^3+^ were found. Moreover, the energy transfer process from Tb^3+^ to Eu^3+^ was verified by the overlap of luminescence spectra and the variation of lifetime. Energy transfer mechanism was determined to be a dipole–dipole interaction, and ET efficiency as well as quantum efficiency were also obtained. Moreover, the emission color of GdSr_2_AlO_5_:Tb^3+^,Eu^3+^ can be tuned from green to red by altering the ratio of Tb^3+^/Eu^3+^. These results indicate that the GdSr_2_AlO_5_:Tb^3+^,Eu^3+^ phosphor is a promising single-component white light-emitting phosphor.

## Abstract 2

The emission spectra of GdSr_2_AlO_5_:2%Tb^3+^,*x*%Eu^3+^ (*x* = 0, 0.5, 1, 2, 3, and 5) under 275 nm excitation. (b) Variation tendency of the green emission of Tb^3+^ and the red emission of Eu^3+^.

## Graphical abstract

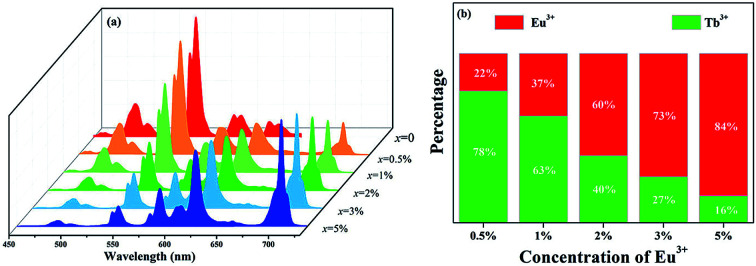

## 1. Introduction

White light-emitting diodes (w-LEDs) with superior advantages, such as high energy efficiency, good physical and chemical stability, long lifetime, and environment friendliness, have been drawing attention and are regarded as the next generation of illumination sources.^1–4^ The most available material for light devices involves Ce^3+^-doped yttrium aluminum garnet (YAG:Ce^3+^) with blue-LED chips, which has been commercialized.^5,6^ Because of the scarcity of red component, it encounters a low color rendering index (*R*_a_ < 80) and a high color temperature (CCT > 7000 K).^7,8^ To overcome these drawbacks, the combination of a UV LED chip with red (Ca_4_(PO_4_)O:Eu^2+^),^9^ green (Na_2_Y_2_B_2_O_7_:Ce^3+^,Tb^3+^),^10^ and blue (Na_*x*_Ca_1−*x*_Al_2−*x*_Si_2+*x*_O_8_:Eu^2+^)^11^ to achieve white-light with high *R*_a_ and appropriate CCT is another approach. However, the mixture of different phosphors brings about an inevitable problem of fluorescence re-absorption, resulting in the loss of luminous efficiency.^12,13^ Therefore, it is obligatory to develop a single-phase white light-emitting phosphor.

Rare earth ion-doped aluminate phosphors, such as LaSr_2_AlO_5_:Ce^3+^/Eu^2+^,^14,15^ (La,Gd)Sr_2_AlO_5_:Ce,^16^ and (La,Gd)Sr_2_(Al,B)O_5_:Ce,^17^ have been studied extensively due to their cheap raw materials and good chemical and physical stability. The structural properties of this aluminate-based phosphor and the effect on the luminescence properties have been investigated. However, the luminescence of Ce^3+^ and Eu^2+^ belong to the f–d transition.^18^ Upon reviewing the literature, it is observed that it is difficult to find other rare-earth ions with f–f transition for doping into the GdSr_2_AlO_5_ phosphor. As is well known, co-doping of different rare ions as a sensitizer and activator plays a significant role in obtaining emission *via* energy transfer processes.^19^

Tb^3+^ and Eu^3+^ with characteristic green^20,21^ and red^22,23^ emissions are widely used in luminescent materials, respectively. Compared to most Eu^3+^-doped phosphors with the dominant emission corresponding to ^5^D_0_ → ^7^F_1_ or ^5^D_0_ → ^7^F_2_, the Eu^3+^-doped GdSr_2_AlO_5_ phosphor shows an intensive emission related to ^5^D_0_ → ^7^F_4_. It is clear that Tb^3+^ functions as a good sensitizer to improve the luminescence efficiency of Eu^3+^ ions in Sr_2_MgSi_2_O_7_,^24^ Na_3_La(PO_4_)_2_,^25^ CaYAlO_4_,^26^ and Y_2_O_3_ (ref. 27) phosphors. In this study, a single-phased GdSr_2_AlO_5_:Tb^3+^,Eu^3+^ phosphor was synthesized by a conventional solid-state method. The efficient energy transfer from Tb^3+^ to Eu^3+^ was systematically investigated with steady state fluorescence, lifetime measurement, and the energy transfer efficiency. Moreover, the color of the total emission can be tuned by altering the ratio of Tb^3+^/Eu^3+^ in the GdSr_2_AlO_5_ host.

## 2. Experimental method

A series of phosphors with the composition of GdSr_2_AlO_5_:Tb^3+^, GdSr_2_AlO_5_:Eu^3+^, and GdSr_2_AlO_5_:Tb^3+^,Eu^3+^ were prepared by a conventional high temperature solid-state method under a reductive atmosphere (5% H_2_ + 95% N_2_) at 1500 °C. SrCO_3_ (A. R.), Al_2_O_3_ (A. R.), Gd_2_O_3_ (A. R.), Tb_4_O_7_ (99.99%), and Eu_2_O_3_ (99.99%) were used as raw materials. They were stoichiometrically weighed and mixed by grinding in an agate mortar for 30 min. After this, they were transferred into ceramic crucibles and calcined in a high-temperature tubular furnace at 1500 °C for 4 h at a heating rate of 3 °C min^−1^. Finally, the samples were cooled down to room temperature in the furnace and ground into powders for subsequent use.

Bruker D8 (voltage 40 kV and current 40 mA) over the 2*θ* range from 10° to 70° with CuKα radiation (*λ* = 1.54178 Å) was used to obtain the XRD patterns of GdSr_2_AlO_5_:Tb^3+^,Eu^3+^ samples. Hitachi F-7000 spectrophotometers with a 150 W xenon lamp light source were used to obtain the excitation and emission spectra. The morphology of the prepared phosphor was examined by a field-emission scanning electron microscope (Hitachi S-4800). The decay curves were obtained using the Hamamatsu Quantaurus-Tau C11367 fluorescence spectrophotometer. The chromaticity data were calculated by the CIE1931 software. Photoluminescence absolute quantum efficiency (QE) was determined *via* Hamamatsu Quantaurus-QY C11347 using an integrated sphere. All the measurements were conducted at room temperature.

## 3. Results and discussion

### 3.1 XRD analysis

**Fig. 1:**
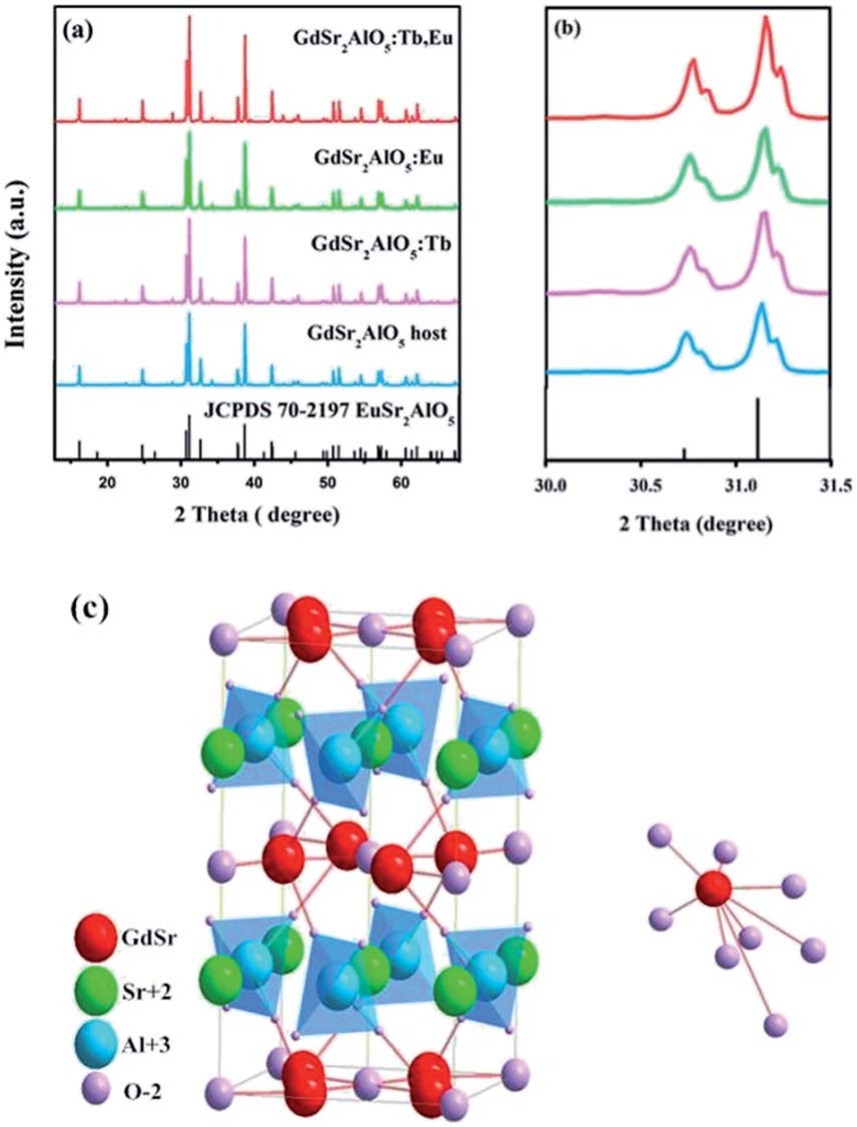
(a) Representative XRD patterns of the samples. (b) Magnified XRD patterns between 30° and 31.5°. (c) Crystal structure of the GdSr_2_AlO_5_ and the coordination environment of GdO_8_.

Fig. 1(a) shows the XRD patterns of the as-prepared GdSr_2_AlO_5_ host and Tb^3+^, Eu^3+^-doped phosphor as well as the standard patterns of EuSr_2_AlO_5_ (JCPDS 70-2197). The patterns agreed with the crystallographic structure of the JCPDS card 70-2197, and impurity peaks were not found. This result illustrates that the obtained samples are in single phase, and doping of Tb^3+^ and/or Eu^3+^ ions at low concentrations does not change the crystal structure. In addition, it can be observed from Fig. 1(b) that the diffraction peak positions of the samples are shifted slightly to a higher angle as compared to those of the standard patterns of EuSr_2_AlO_5_ (JCPDS 70-2197); this can be due to the replacement of large-size Eu^3+^ sites by the smaller-size trivalent ions. Hence, RE^3+^ ions were effectively doped into the lattice; this could be confirmed by the slight change in the diffraction peaks.

Fig. 1(c) displays a partial structure of GdSr_2_AlO_5_. As reported previously, the crystal structure of GdSr_2_AlO_5_ is isostructural with the standard file of EuSr_2_AlO_5_ (JCPDS no. 70-2197, tetragonal, space group *I*4/*mcm*).^28^ Gd atoms will occupy the half 8h sites, and the other half is occupied by the Sr atoms. The cell also contains 4a sites, which are fully occupied by the Sr atoms, and the Al atoms will occupy the 4b sites. The 4c sites and 16l sites are occupied by O, denoted as O_1_ and O_2_, respectively. The GdO_8_ polyhedron consists of two O_1_ atoms and six O_2_ atoms, where the latter are shared by four AlO_4_ tetrahedral on the adjacent layers, forming an isolated structure. Fortunately, isolation in space weakens the interaction between the paired ions. To some extent, the concentrating quenching effect would be avoided.^29^

### 3.2 SEM with energy dispersive X-ray analysis

**Fig. 2:**
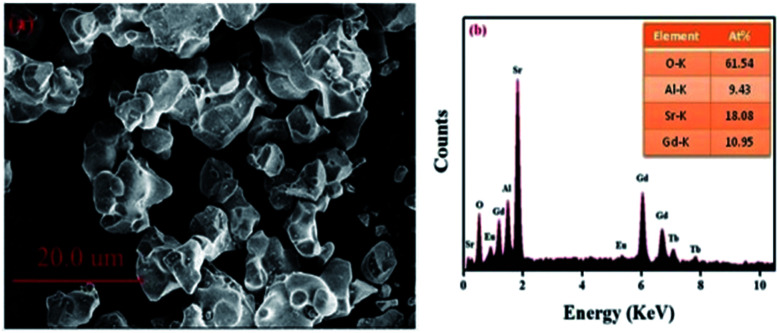
SEM image (a) and EDS spectrum (b) of GdSr_2_AlO_5_:2%Tb^3+^,2%Eu^3+^.

Fig. 2(a) displays the morphology of the GdSr_2_AlO_5_:Tb^3+^,Eu^3+^ sample. The surface image shows irregular particles with a size range of 5–10 μm. Moreover, as shown in Fig. 2(b), EDS is used to analyze the chemical composition of the GdSr_2_AlO_5_:Tb^3+^,Eu^3+^ phosphor, and the result confirms the presence of Gd, Sr, Al, O, Tb, and Eu elements in the phosphor. Compared with the abovementioned XRD results, the EDS results demonstrate that Tb/Eu ions have been successfully incorporated into the GdSr_2_AlO_5_ lattice.

### 3.3 Luminescence properties

**Fig. 3:**
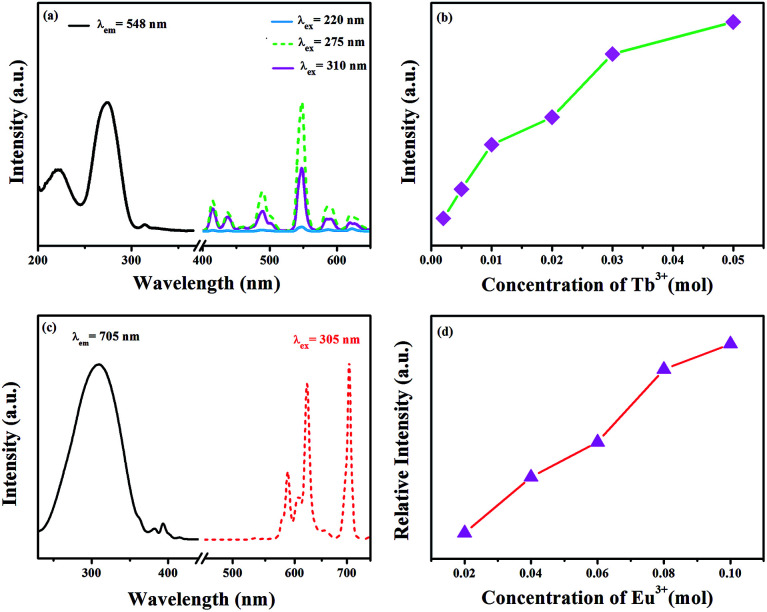
(a) Excitation and emission spectra of the GdSr_2_AlO_5_:2%Tb^3+^ phosphor. (b) Concentration-dependent luminescence intensity of GdSr_2_AlO_5_:*x*Tb^3+^. (c) Excitation and emission spectra of the GdSr_2_AlO_5_:2%Eu^3+^ phosphor and (d) concentration-dependent luminescence intensity of GdSr_2_AlO_5_:*y*Eu^3+^.

The excitation spectrum of GdSr_2_AlO_5_:2%Tb^3+^ is shown in Fig. 3(a). The excitation spectrum monitored at 548 nm exhibits a broad band and several weak peaks. The broad band from 200 nm to 250 nm centered at 220 nm is ascribed to the f–d transitions of the Tb^3+^ ions, whereas the excitation peaks at 275 and 310 nm originate from the transitions of ^8^S_7/2_ → ^6^I_7/2_ and ^8^S_7/2_ → ^6^P_7/2_ of the Gd^3+^ ions, respectively.^30,31^ The appearance of strong excitation sharp bands of Gd^3+^ ions confirms that there is an efficient energy transfer from Gd^3+^ to Tb^3+^. The emission spectra of the phosphor under 220, 275, and 310 nm excitation in the wavelength range from 400–650 nm are also depicted in Fig. 3(a). There are two groups of lines in the emission spectrum: one group in the region from 400 to 480 nm is derived from the ^5^D_3_ → ^7^F_*J*_ (*J* = 5, 4, and 3) transitions of Tb^3+^. The second groups of lines in the wavelength range from 480 to 650 nm originate from the ^5^D_4_ → ^7^F_*J*_ (*J* = 6, 5, 4, and 3) transitions. Among these emissions, the ^5^D_4_ → ^7^F_5_ transition with a maximum at about 548 nm exhibits the strongest emission intensity. The emission intensity increases with the increasing concentration of Tb^3+^, as presented in Fig. 3(b). Concentration quenching does not occur in GdSr_2_AlO_5_:Tb^3+^.

Fig. 3(c) and (d) present the excitation and emission spectra as well as concentration-dependent luminescence intensity of GdSr_2_AlO_5_:*y*Eu^3+^. The excitation spectrum of the Eu^3+^-activated sample monitoring the emission wavelength at 705 nm contains a broad band in the wavelength range from 220 to 350 nm, corresponding to the charge transfer transition of Eu^3+^ (O^2−^ → Eu^3+^) and several characteristic narrow peaks at 383 nm and 393 nm. They are attributed to ^7^F_0_ → ^5^G_2_ and ^7^F_0_ → ^5^L_6_ electronic transitions of the Eu^3+^ ions. In this band, there should be absorption of transitions from ^8^S_7/2_ to ^6^I_7/2_ level of Gd^3+^, which is too weak and is overlapped with the strong CTB of the O^2−^ → Eu^3+^.^32,33^ The emission spectrum consists of multiple band emissions at 587, 607, 622, 657, and 705 nm, which are assigned to the ^5^D_0_ → ^7^F_*J*_ (*J* = 0, 1, 2, 3, and 4) transitions of Eu^3+^ ions,^34^ respectively. Among these emission peaks, the two most intense lines are the emissions located at 622 and 705 nm. Obviously, the magnetic dipole transition of ^5^D_0_ → ^7^F_1_ is stronger than the ^5^D_0_ → ^7^F_2_ electronic transition; this indicates that Eu^3+^ ions in the GdSr_2_AlO_5_ host occupy a site with a non-inversion symmetry; this is consistent with its structure. Compared to other Eu^3+^ phosphors, GdSr_2_AlO_5_:Eu^3+^ provides more red components to improve *R*_a_. Similarly, there is no evident concentration quenching with the change in the Eu^3+^ concentration.

**Fig. 4:**
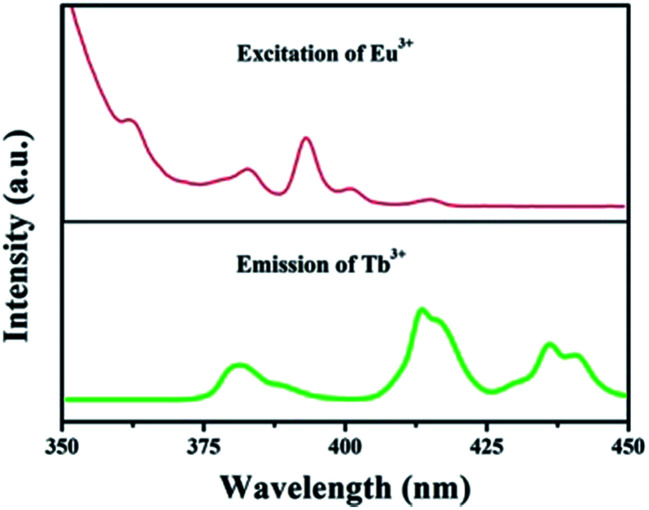
Spectral overlapping of the PL spectrum of GdSr_2_AlO_5_:Tb^3+^ and the PLE spectrum of GdSr_2_AlO_5_:Eu^3+^.

The foundation of energy transfer is the spectral overlapping Tb^3+^ emission and the Eu^3+^ excitation. As is clearly shown in Fig. 4, there are some overlaps between the emission of ^5^D_3_ → ^7^F_6_ (Tb^3+^) and the excitation of ^7^F_0_ → ^5^G_2,3,4,5_, ^5^L_8_ (Eu^3+^). Moreover, the excitation of ^7^F_0_ → ^5^D_3_, ^5^L_6_ (Eu^3+^) overlaps with the emission of ^5^D_3_ → ^7^F_5_ (Tb^3+^).^35^

**Fig. 5:**
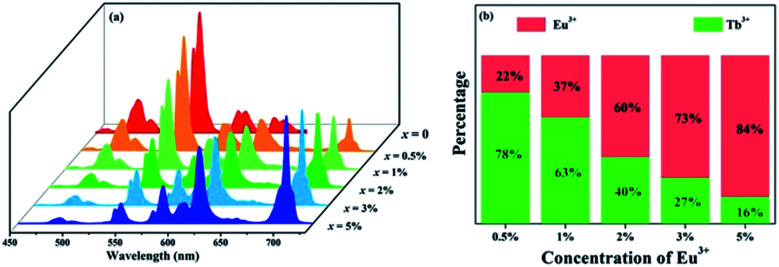
(a) Emission spectra of GdSr_2_AlO_5_:2%Tb^3+^,*x*%Eu^3+^ (*x* = 0, 0.5, 1, 2, 3, and 5) under 275 nm excitation. (b) Variation tendency of the green emission of Tb^3+^ and the red emission of Eu^3+^.

To further investigate the energy transfer process, a series of samples of GdSr_2_AlO_5_:2%Tb^3+^,*x*%Eu^3+^ (*x* = 0, 0.5, 1, 2, 3, and 5) were prepared whose emission spectra under the excitation at 275 nm are presented in Fig. 5(a). The characteristic sharp emission peaks of Tb^3+^ and Eu^3+^ are observed. The tendency in the variation of Tb^3+^ and Eu^3+^ differs significantly. The emission intensity of Tb^3+^ at 548 nm decreases remarkably with an increase in the concentration of Eu^3+^, whereas the emission intensity of Eu^3+^ increases monotonously. Fig. 5(b) depicts the contribution of different ions in the corresponding emission spectra. The G/R (green to red) ratio continuously decreases notably. Based on the prerequisite of the concentration of Tb^3+^ being a constant value, explanation for this phenomenon should be the evidence of energy transfer from Tb^3+^ to Eu^3+^.

**Fig. 6:**
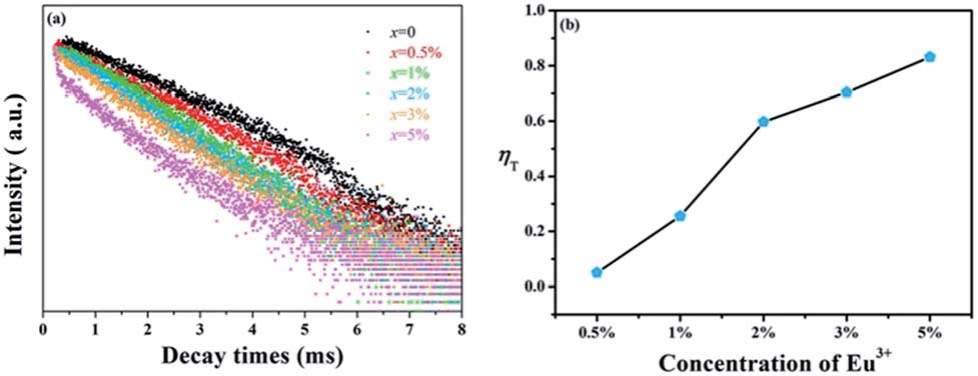
(a) Decay curves of Tb^3+^ in GdSr_2_AlO_5_:2%Tb^3+^,*x*%Eu^3+^ (*x* = 0, 0.5, 1, 2, 3, and 5). (b) Energy transfer efficiencies (*η*_T_) in GdSr_2_AlO_5_:2%Tb^3+^,*x*%Eu^3+^ as a function of different Eu^3+^ concentrations.

In spite of steady-state spectra, kinetic spectra are necessary to better analyze the energy transfer mechanism of Tb^3+^ → Eu^3+^ in GdSr_2_AlO_5_. Fig. 6(a) shows the fluorescence decay curves of Tb^3+^ emissions, which are obtained under 275 nm excitation and detected at 548 nm. The luminescence curves can be fitted well with the double-exponential expression:^36,37^

1 *I*(*t*) = *I*_0_ + *A*_1_ exp(−*t*/*τ*_1_) + *A*_2_ exp(−*t*/*τ*_2_)

where *I* and *I*_0_ represent the luminescence intensity at time *t* and 0, *A*_1_ and *A*_2_ are equal to constants, *t* is the time, and *τ*_1_ and *τ*_2_ are the rapid and slow lifetimes for exponential components, respectively. As a function of these parameters, the average luminescence lifetime (*τ*) was determined by the following equation:

2 *τ* = (*A*_1_*τ*_1_^2^ + *A*_2_*τ*_2_^2^)/(*A*_1_*τ*_1_ + *A*_2_*τ*_2_)

The values of *τ* are calculated to decrease when the Eu^3+^ concentration increases; this indicates that energy transfer occurs from Tb^3+^ to Eu^3+^, as expected. Furthermore, the energy transfer efficiency is calculated by the expression as follows:^38^

3 *η*_T_ = 1 − *I*_s_/*I*_so_

where *η*_T_ represents the energy transfer efficiency, and *I*_s_ and *I*_so_ represent the luminescence intensity of Tb^3+^ in the presence and absence of Eu^3+^, respectively. The results of *η*_T_ are shown in Fig. 6(b). It can be found that the efficiencies from Tb^3+^ to Eu^3+^ increase with the increasing Eu^3+^ concentration, which can reach the maximum value of 83%. Therefore, the energy transfer from Tb^3+^ to Eu^3+^ ions is efficient to improve Eu^3+^ luminescence.

### 3.4 Energy transfer mechanism

The type of energy transfer should depend on either the exchange interaction or the multipolar interaction. It relies on the critical distance between the Tb^3+^ (sensitizer) and Eu^3+^ (activator), and the critical distance is expressed by the following formula:^39^4
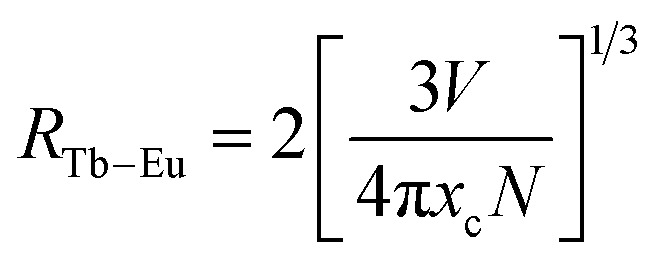
where *V* represents the cell volume, *N* is equal to the number of cations in the unit cell, and *x*_c_ is the total concentration of Tb^3+^ and Eu^3+^. In this case, *N* = 4 and *V* = 498.64 Å^3^. The corresponding distance is calculated to be more than 5 Å, indicating that Tb^3+^ → Eu^3+^ energy transfer would tend to be a multipolar interaction. On account of energy transfer mechanism for multipolar interaction, the following expression can be given:^40,41^

**Fig. 7:**
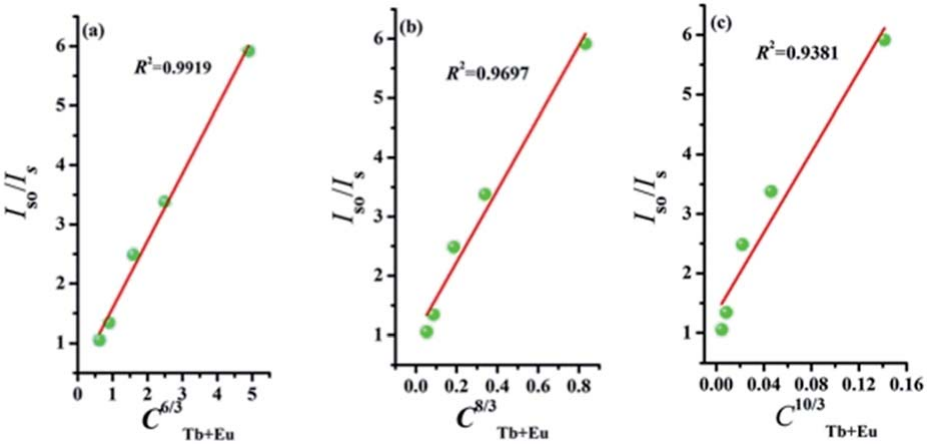
Dependence of *I*_so_/*I*_s_ of Tb^3+^ on (a) *C*^6/3^, (b) *C*^8/3^, and (c) *C*^10/3^.

5 *I*_so_/*I*_s_ ∝ *C*^*n*/3^

where *I*_so_ and *I*_s_ are the luminescence intensity of the sensitizer without and with activator. *C* represents the doping concentration of Tb^3+^ and Eu^3+^ ion. The values for *n* = 6, 8, and 10 correspond to dipole–dipole, dipole–quadrupole, and quadrupole–quadrupole interactions. Results are illustrated in Fig. 7. The best linear fit was obtained when *n* = 6, which confirmed energy transfer through dipole–dipole mechanism.

**Fig. 8:**
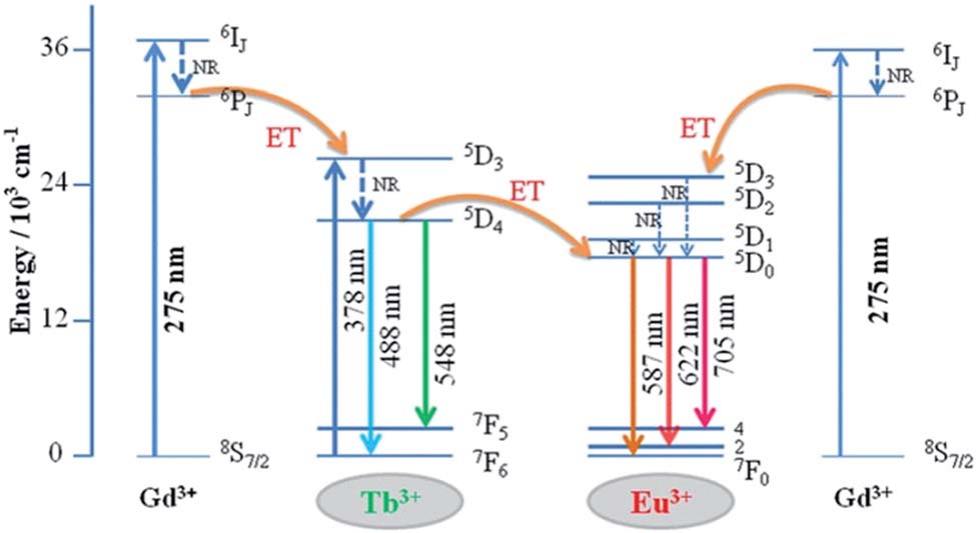
Schematic of energy transfer from Tb^3+^ to Eu^3+^ in GdSr_2_AlO_5_.

The detailed Gd^3+^ → Tb^3+^, Gd^3+^ → Eu^3+^, and Tb^3+^ → Eu^3+^ energy transfer processes are shown in Fig. 8. The electrons were excited from the ^8^S_7/2_ ground state to the ^6^I_J_ excited state of Gd^3+^ under 275 nm excitation. Because the energy levels ^5^D_3_ of Tb^3+^ and ^5^D_1_ state of Eu^3+^ are lower than the ^6^I_*J*_ state of Gd^3+^ in the diagram, the energy transfer processes from Gd^3+^ → Tb^3+^ and Gd^3+^ → Eu^3+^ occur simultaneously. In addition, the electrons absorbing energy were excited from the ^7^F_6_ ground state to the ^5^D_3_ excited state of Tb^3+^. The energy transfer process occurs between Tb^3+^ and Eu^3+^ activators since the ^5^D_3_ state of Tb^3+^ lies higher than the ^5^D_1_ state of Eu^3+^. Then, the electrons from the excited state of ^5^D_3_ (Tb^3+^) and ^5^D_1_ (Eu^3+^) relaxed to ^5^D_4_ and ^5^D_0_, respectively. Finally, green to red emission is yielded by electrons returning to their respective ground states.

### 3.5 CIE chromaticity coordinate and QE

**Fig. 9:**
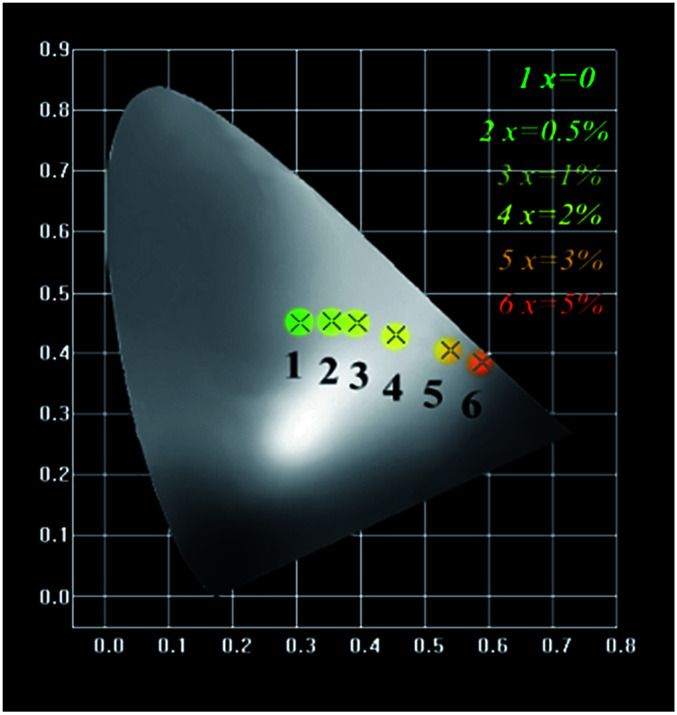
CIE chromaticity diagram for GdSr_2_AlO_5_:2%Tb^3+^,*x*%Eu^3+^ (*x* = 0, 0.5, 1, 2, 3, and 5) samples.

The Commission Internationale de L'Eclairage (CIE 1931) chromaticity coordinates of GdSr_2_AlO_5_:2%Tb^3+^,*x*%Eu^3+^ phosphors under the excitation at 275 nm are presented in Fig. 9. The CIE coordinates shifted from (0.320, 0.466) to (0.574, 0.403) for the samples *x* = 0 to *x* = 5% correspond to green, yellow-green, and red. It is clear that the coordinate *Y* changes linearly with the *X*, the expression of which can be shown as below:

6 *Y* = 0.7541 − 0.6109*X*

The equation proves that the range of chromaticity coordinates can be obtained by adjusting the ratio of Tb^3+^ and Eu^3+^ mathematically. The as-prepared phosphor emits bright tunable visible light, implying its potential application in multicolor displays, and meets the requirement for application in *n*-UV LEDs.

**Fig. 10:**
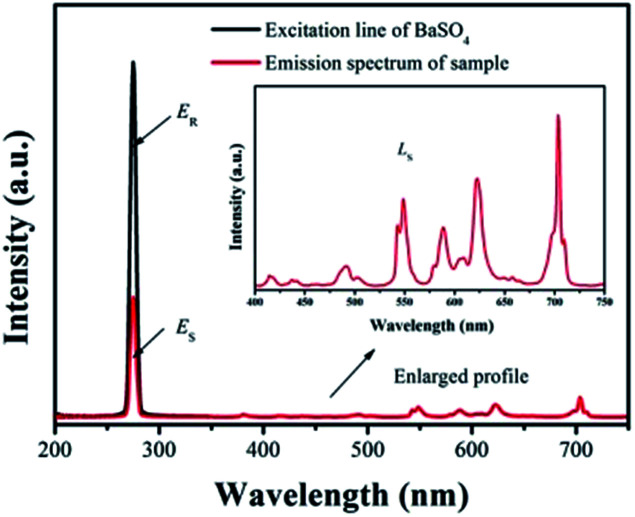
Excitation line of BaSO_4_ and emission spectrum of the sample. Inset shows the magnification of the emission spectrum.

For photo-luminescence application, the importance of quantum efficiency (QE) should be considered. According to the method described by De Mello *et al.*, QE can be calculated by the following equation:^42^7
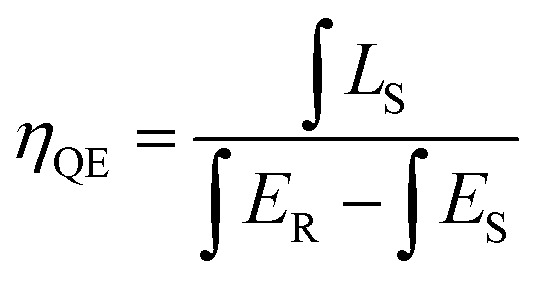
where *L*_S_ is the emission of the sample, *E*_S_ equals to the spectrum of the sample excited by the light, and *E*_R_ represents the spectrum of the excitation light without the sample. The results are listed in Fig. 10. Under 275 nm sexcitation, the calculated QE of GdSr_2_AlO_5_:2%Tb^3+^,2%Eu^3+^ is 78.5%, which demonstrates a high QE.

## 4. Conclusions

The GdSr_2_AlO_5_:Tb^3+^,Eu^3+^ phosphors were prepared by a high temperature solid-state reaction method, and their luminescence properties were investigated in detail. Due to REO_8_ structure isolation, damping of the concentration quenching would occur. Energy transfer from Gd^3+^ → Eu^3+^ and Gd^3+^ → Tb^3+^ was found in the excitation spectrum. Moreover, the luminescence properties and the transient spectra confirmed the efficiency energy transfer from Tb^3+^ to Eu^3+^. The mechanism it demonstrated is dipole–dipole interaction. GdSr_2_AlO_5_:Tb^3+^,Eu^3+^ phosphors were able to provide multi-color emission from green, yellow, orange, and finally to deep red by the different ratios of Tb^3+^/Eu^3+^. Additionally, the QE can reach about 78.5%. All the results indicate that the series of GdSr_2_AlO_5_:Tb^3+^,Eu^3+^ phosphors can be a candidate for color tunable luminescence materials used for *n*-UV LEDs.

## Conflicts of interest

There are no conflicts to declare.

## Supplementary Material
